# The cocrystal 2-hydr­oxy-4-methyl-*N*-propanoylbenzohydrazide–2-hydr­oxy-*N*-(2-hydr­oxy-4-methyl­benzo­yl)-6-methyl­benzohydrazide (2/1)

**DOI:** 10.1107/S1600536808033515

**Published:** 2008-10-18

**Authors:** Hai-Mei Feng, Xin Wang, Ke-Wei Lei

**Affiliations:** a State Key Laboratory Base of Novel Functional Materials and Preparation Science, Institute of Solid Materials Chemistry, Faculty of Materials Science and Chemical Engineering, Ningbo University, Ningbo 315211, People’s Republic of China; b Zhejiang Textile and Fashion College, Ningbo 315211, People’s Republic of China

## Abstract

The asymmetric unit of the title compound, 2C_11_H_14_N_2_O_3_·C_16_H_16_N_2_O_4_, contains one mol­ecule of 2-hydr­oxy-4-methyl-*N*-propanoylbenzohydrazide and one-half of a mol­ecule of 2-hydr­oxy-*N*-(2-hydr­oxy-4-methyl­benzo­yl)-6-methyl­benzohydrazide. The latter is located on a centre of inversion. Intra­molecular N—H⋯O inter­actions stabilize the conformations of both mol­ecules. The crystal structure is stabilized by inter­molecular N—H⋯O and O—H⋯O hydrogen bonds.

## Related literature

For related literature, see: John *et al.* (2007 ▶); Majumder *et al.* (2006 ▶).
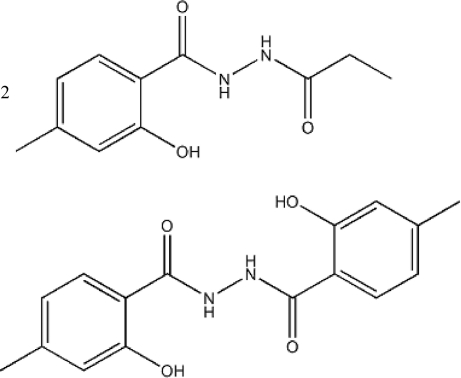


## Experimental

### 

#### Crystal data

2C_11_H_14_N_2_O_3_·C_16_H_16_N_2_O_4_
*M* *_r_* = 744.79
Triclinic, 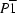

*a* = 6.5778 (10) Å
*b* = 10.7618 (17) Å
*c* = 13.936 (2) Å
α = 109.522 (3)°
β = 93.608 (1)°
γ = 104.448 (4)°
*V* = 888.8 (2) Å^3^
*Z* = 1
Mo *K*α radiation
μ = 0.10 mm^−1^
*T* = 296 (2) K
0.54 × 0.30 × 0.25 mm

#### Data collection

Bruker APEXII diffractometer
Absorption correction: multi-scan (*SADABS*; Bruker, 2005 ▶) *T*_min_ = 0.964, *T*_max_ = 0.975
5076 measured reflections
4125 independent reflections
1865 reflections with *I* > 2σ(*I*)
*R*_int_ = 0.022

#### Refinement

*R*[*F* ^2^ > 2σ(*F* ^2^)] = 0.037
*wR*(*F* ^2^) = 0.110
*S* = 0.90
4125 reflections
246 parameters
H atoms treated by a mixture of independent and constrained refinement
Δρ_max_ = 0.20 e Å^−3^
Δρ_min_ = −0.22 e Å^−3^

### 

Data collection: *APEX2* (Bruker, 2007 ▶); cell refinement: *SAINT* (Bruker, 2007 ▶); data reduction: *SAINT*; program(s) used to solve structure: *SHELXS97* (Sheldrick, 2008 ▶); program(s) used to refine structure: *SHELXL97* (Sheldrick, 2008 ▶); molecular graphics: *SHELXTL* (Sheldrick, 2008 ▶); software used to prepare material for publication: *SHELXTL*.

## Supplementary Material

Crystal structure: contains datablocks global, I. DOI: 10.1107/S1600536808033515/bt2793sup1.cif

Structure factors: contains datablocks I. DOI: 10.1107/S1600536808033515/bt2793Isup2.hkl

Additional supplementary materials:  crystallographic information; 3D view; checkCIF report

## Figures and Tables

**Table 1:** Hydrogen-bond geometry (Å, °)

*D*—H⋯*A*	*D*—H	H⋯*A*	*D*⋯*A*	*D*—H⋯*A*
N1—H1*D*⋯O1	0.86	1.93	2.620 (2)	136
O1—H1*E*⋯O4	0.82	1.87	2.682 (2)	169
N2—H2*A*⋯O2^i^	0.86	2.03	2.866 (2)	165
N3—H3*B*⋯O5	0.84 (2)	1.94 (3)	2.613 (3)	136 (2)
N3—H3*B*⋯O4^ii^	0.84 (2)	2.37 (3)	2.655 (3)	101 (2)
O5—H5*B*⋯O3^ii^	0.89 (3)	1.80 (3)	2.685 (2)	175 (2)

Symmetry codes: (i) 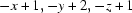; (ii) 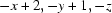.

**Fig. 1.**
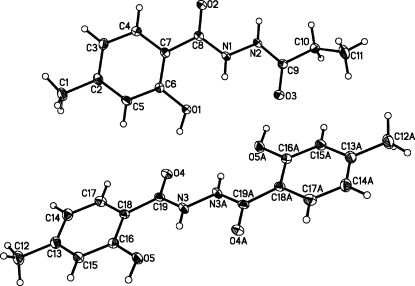
The structure of (I), showing 30% probability displacement ellipsoids and the atom-numbering scheme [symmetry code: (A) -x + 2, -y + 1, -z].

**Fig. 2.**
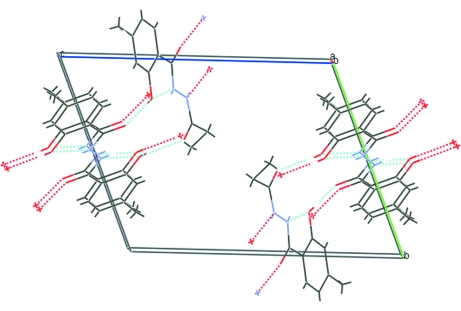
Packing diagram of the title compound.

## supplementary crystallographic information

### Comment

*N*-Acylsalicylhydrazides are an interesting class of compounds because of their unique properties. They have been used extensively as ligands in coordination chemistry. *N*-acylsalicylhydrazide compounds show photoluminescence in the solid state by proton transfer from O atom to the imine N atom (Majumder *et al.*, 2006). The nuclearity and the shape of the metallamacrocycles could be modulated by controlling the steric interactions caused by *N*-acyl tails of the ligands (John *et al.*, 2007).

A view of the title structure is illustrated in Fig. 1. The asymmetric unit contains one molecule of 2-hydroxy-4-methyl-*N*-propanoylbenzohydrazide and half a molecule of 2-hydroxy-*N*-(2-hydroxy-4-methylbenzoyl)-6-methylbenzohydrazide.

The molecular conformation is characterized by N—H···O hydrogen bonds and the crystal packing is stabilized by N—H···O and O—H···O hydrogen bonds (Fig. 2).

### Experimental

Propionic anhydride (0.26 g, 2.00 mmol) and 2-hydroxy-4-methylbenzohydrazide (0.31 g, 1.80 mmol) were stirred with an external ice-water bath in DMF (20 ml) for 6 h. The filtrate was evaporated on a rotary evaporator. After recrystallization, the title compound were obtained.

### Refinement

All H atoms were placed in geometrically idealized positions and constrained to ride on their parent atoms (C—H = 0.93 Å; N—H = 0.86 Å; O—H = 0.82 Å) and *U*_iso_(H) values were set to 1.2*U*_eq_(C, N) and 1.5Ueq(O).

### Crystal data

**Table:**

2C_11_H_14_N_2_O_3_·C_16_H_16_N_2_O_4_	*Z* = 1
*M**_r_* = 744.79	*F*(000) = 394
Triclinic, *P*1	*D*_x_ = 1.391 Mg m^−^^3^
Hall symbol: -P 1	Melting point = 488–496 K
*a* = 6.5778 (10) Å	Mo *K*α radiation, λ = 0.71073 Å
*b* = 10.7618 (17) Å	Cell parameters from 6530 reflections
*c* = 13.936 (2) Å	θ = 1.0–27.6°
α = 109.522 (3)°	µ = 0.10 mm^−^^1^
β = 93.608 (1)°	*T* = 296 K
γ = 104.448 (4)°	Block, colourless
*V* = 888.8 (2) Å^3^	0.54 × 0.30 × 0.25 mm

### Data collection

**Table:**

Bruker APEXII diffractometer	4125 independent reflections
Radiation source: fine-focus sealed tube	1865 reflections with *I* > 2σ(*I*)
graphite	*R*_int_ = 0.022
Detector resolution: 0 pixels mm^-1^	θ_max_ = 27.6°, θ_min_ = 1.0°
ω scans	*h* = −8→8
Absorption correction: multi-scan (SADABS; Bruker, 2005)	*k* = −14→14
*T*_min_ = 0.964, *T*_max_ = 0.975	*l* = −18→18
5076 measured reflections	

### Refinement

**Table:**

Refinement on *F*^2^	Primary atom site location: structure-invariant direct methods
Least-squares matrix: full	Secondary atom site location: difference Fourier map
*R*[*F*^2^ > 2σ(*F*^2^)] = 0.037	Hydrogen site location: inferred from neighbouring sites
*wR*(*F*^2^) = 0.110	H atoms treated by a mixture of independent and constrained refinement
*S* = 0.90	*w* = 1/[σ^2^(*F*_o_^2^) + (0.0665*P*)^2^ + 0.5716*P*] where *P* = (*F*_o_^2^ + 2*F*_c_^2^)/3
4125 reflections	(Δ/σ)_max_ = 0.001
246 parameters	Δρ_max_ = 0.20 e Å^−^^3^
0 restraints	Δρ_min_ = −0.22 e Å^−^^3^

### Special details

**Table:**

Geometry. All e.s.d.'s (except the e.s.d. in the dihedral angle between two l.s. planes) are estimated using the full covariance matrix. The cell e.s.d.'s are taken into account individually in the estimation of e.s.d.'s in distances, angles and torsion angles; correlations between e.s.d.'s in cell parameters are only used when they are defined by crystal symmetry. An approximate (isotropic) treatment of cell e.s.d.'s is used for estimating e.s.d.'s involving l.s. planes.
Refinement. Refinement of *F*^2^ against ALL reflections. The weighted *R*-factor *wR* and goodness of fit *S* are based on *F*^2^, conventional *R*-factors *R* are based on *F*, with *F* set to zero for negative *F*^2^. The threshold expression of *F*^2^ > σ(*F*^2^) is used only for calculating *R*-factors(gt) *etc*. and is not relevant to the choice of reflections for refinement. *R*-factors based on *F*^2^ are statistically about twice as large as those based on *F*, and *R*- factors based on ALL data will be even larger.

### Fractional atomic coordinates and isotropic or equivalent isotropic displacement parameters (Å^2^)

**Table:**

	*x*	*y*	*z*	*U*_iso_*/*U*_eq_	
C1	1.5753 (4)	1.1759 (2)	0.25135 (19)	0.0371 (6)	
H1A	1.6241	1.2745	0.2819	0.056*	
H1B	1.5448	1.1483	0.1777	0.056*	
H1C	1.6836	1.1382	0.2689	0.056*	
C2	1.3767 (3)	1.1237 (2)	0.29126 (16)	0.0272 (5)	
C3	1.2717 (3)	1.2119 (2)	0.35009 (16)	0.0302 (5)	
H3A	1.3247	1.3062	0.3659	0.036*	
C4	1.0896 (3)	1.1610 (2)	0.38542 (16)	0.0272 (5)	
H4A	1.0227	1.2222	0.4251	0.033*	
C5	1.2941 (3)	0.9840 (2)	0.27002 (16)	0.0271 (5)	
H5A	1.3639	0.9236	0.2317	0.032*	
C6	1.1100 (3)	0.9315 (2)	0.30440 (15)	0.0243 (5)	
C7	1.0027 (3)	1.0199 (2)	0.36312 (15)	0.0230 (5)	
C8	0.8034 (3)	0.9769 (2)	0.40407 (15)	0.0230 (5)	
C9	0.4670 (3)	0.6593 (2)	0.40029 (15)	0.0272 (5)	
C10	0.2783 (3)	0.6153 (2)	0.44886 (17)	0.0356 (6)	
H10A	0.2494	0.6963	0.4956	0.043*	
H10B	0.3117	0.5625	0.4890	0.043*	
C11	0.0800 (4)	0.5283 (3)	0.3690 (2)	0.0434 (6)	
H11A	−0.0362	0.5022	0.4034	0.065*	
H11B	0.1071	0.4472	0.3232	0.065*	
H11C	0.0442	0.5809	0.3303	0.065*	
C12	2.0539 (4)	0.8165 (3)	−0.0799 (2)	0.0423 (6)	
H12A	2.0282	0.8592	−0.1281	0.063*	
H12B	2.1120	0.7422	−0.1122	0.063*	
H12C	2.1528	0.8831	−0.0208	0.063*	
C13	1.8475 (3)	0.7613 (2)	−0.04652 (17)	0.0312 (5)	
C14	1.8192 (3)	0.8096 (2)	0.05601 (17)	0.0331 (5)	
H14A	1.9293	0.8769	0.1054	0.040*	
C15	1.6810 (3)	0.6616 (2)	−0.11824 (16)	0.0293 (5)	
H15A	1.6985	0.6282	−0.1870	0.035*	
C16	1.4884 (3)	0.6101 (2)	−0.08999 (16)	0.0267 (5)	
C17	1.6287 (3)	0.7582 (2)	0.08499 (17)	0.0320 (5)	
H17A	1.6136	0.7910	0.1541	0.038*	
C18	1.4587 (3)	0.6587 (2)	0.01368 (16)	0.0257 (5)	
C19	1.2600 (3)	0.6131 (2)	0.05327 (16)	0.0252 (5)	
N1	0.7128 (3)	0.84114 (17)	0.37670 (13)	0.0273 (4)	
H1D	0.7663	0.7838	0.3347	0.033*	
N2	0.5341 (3)	0.79291 (17)	0.41560 (13)	0.0263 (4)	
H2A	0.4672	0.8484	0.4492	0.032*	
O4	1.2460 (2)	0.65694 (15)	0.14648 (11)	0.0333 (4)	
O1	1.0312 (2)	0.79309 (14)	0.28105 (11)	0.0322 (4)	
H1E	1.1084	0.7533	0.2467	0.048*	
O2	0.7227 (2)	1.06102 (14)	0.45964 (11)	0.0314 (4)	
O3	0.5567 (2)	0.57635 (14)	0.34881 (11)	0.0329 (4)	
N3	1.0943 (3)	0.52334 (18)	−0.01587 (15)	0.0291 (5)	
O5	1.3246 (2)	0.51267 (15)	−0.16289 (12)	0.0333 (4)	
H3B	1.104 (4)	0.490 (2)	−0.0786 (19)	0.036 (7)*	
H5B	1.368 (4)	0.481 (3)	−0.223 (2)	0.055 (8)*	

### Atomic displacement parameters (Å^2^)

**Table:**

	*U*^11^	*U*^22^	*U*^33^	*U*^12^	*U*^13^	*U*^23^
C1	0.0265 (12)	0.0416 (14)	0.0485 (14)	0.0077 (11)	0.0136 (10)	0.0233 (11)
C2	0.0192 (11)	0.0345 (13)	0.0299 (12)	0.0067 (10)	0.0031 (9)	0.0148 (10)
C3	0.0249 (12)	0.0268 (12)	0.0401 (13)	0.0045 (10)	0.0048 (10)	0.0158 (10)
C4	0.0249 (12)	0.0262 (12)	0.0316 (12)	0.0094 (10)	0.0067 (9)	0.0100 (9)
C5	0.0226 (12)	0.0307 (12)	0.0293 (12)	0.0108 (10)	0.0085 (9)	0.0096 (9)
C6	0.0235 (12)	0.0234 (12)	0.0256 (11)	0.0058 (9)	0.0056 (9)	0.0089 (9)
C7	0.0196 (11)	0.0280 (12)	0.0221 (11)	0.0073 (9)	0.0038 (8)	0.0095 (9)
C8	0.0208 (11)	0.0267 (12)	0.0222 (11)	0.0079 (10)	0.0042 (9)	0.0087 (9)
C9	0.0287 (12)	0.0254 (12)	0.0253 (11)	0.0071 (10)	0.0090 (9)	0.0060 (9)
C10	0.0349 (13)	0.0255 (12)	0.0442 (14)	0.0059 (10)	0.0223 (11)	0.0088 (10)
C11	0.0267 (13)	0.0530 (15)	0.0628 (17)	0.0105 (12)	0.0157 (12)	0.0356 (13)
C12	0.0302 (13)	0.0454 (14)	0.0524 (15)	0.0062 (11)	0.0121 (11)	0.0216 (12)
C13	0.0278 (12)	0.0308 (12)	0.0407 (14)	0.0104 (10)	0.0087 (10)	0.0182 (11)
C14	0.0267 (13)	0.0323 (13)	0.0373 (13)	0.0046 (10)	0.0022 (10)	0.0122 (10)
C15	0.0303 (13)	0.0303 (12)	0.0302 (12)	0.0104 (10)	0.0108 (10)	0.0123 (10)
C16	0.0259 (12)	0.0248 (11)	0.0302 (12)	0.0090 (9)	0.0061 (9)	0.0093 (9)
C17	0.0333 (13)	0.0346 (13)	0.0283 (12)	0.0126 (11)	0.0076 (10)	0.0091 (10)
C18	0.0276 (12)	0.0233 (11)	0.0313 (12)	0.0117 (9)	0.0119 (10)	0.0120 (9)
C19	0.0287 (13)	0.0233 (11)	0.0303 (13)	0.0143 (10)	0.0126 (10)	0.0117 (10)
N1	0.0245 (10)	0.0254 (10)	0.0316 (10)	0.0066 (8)	0.0167 (8)	0.0079 (8)
N2	0.0222 (10)	0.0266 (10)	0.0321 (10)	0.0089 (8)	0.0167 (8)	0.0097 (8)
O4	0.0351 (9)	0.0339 (9)	0.0309 (9)	0.0122 (7)	0.0152 (7)	0.0083 (7)
O1	0.0301 (9)	0.0232 (8)	0.0429 (9)	0.0079 (7)	0.0195 (7)	0.0087 (7)
O2	0.0284 (8)	0.0286 (8)	0.0383 (9)	0.0105 (7)	0.0155 (7)	0.0101 (7)
O3	0.0338 (9)	0.0282 (8)	0.0356 (9)	0.0094 (7)	0.0166 (7)	0.0076 (7)
N3	0.0258 (10)	0.0340 (11)	0.0290 (11)	0.0080 (9)	0.0153 (9)	0.0116 (9)
O5	0.0280 (9)	0.0366 (9)	0.0262 (9)	0.0017 (7)	0.0106 (7)	0.0044 (7)

### Geometric parameters (Å, °)

**Table:**

C1—C2	1.505 (3)	C11—H11C	0.9600
C1—H1A	0.9600	C12—C13	1.508 (3)
C1—H1B	0.9600	C12—H12A	0.9600
C1—H1C	0.9600	C12—H12B	0.9600
C2—C5	1.385 (3)	C12—H12C	0.9600
C2—C3	1.388 (3)	C13—C15	1.382 (3)
C3—C4	1.380 (3)	C13—C14	1.390 (3)
C3—H3A	0.9300	C14—C17	1.381 (3)
C4—C7	1.399 (3)	C14—H14A	0.9300
C4—H4A	0.9300	C15—C16	1.387 (3)
C5—C6	1.388 (3)	C15—H15A	0.9300
C5—H5A	0.9300	C16—O5	1.371 (2)
C6—O1	1.365 (2)	C16—C18	1.407 (3)
C6—C7	1.400 (3)	C17—C18	1.391 (3)
C7—C8	1.492 (3)	C17—H17A	0.9300
C8—O2	1.234 (2)	C18—C19	1.485 (3)
C8—N1	1.342 (3)	C19—O4	1.243 (2)
C9—O3	1.244 (2)	C19—N3	1.330 (3)
C9—N2	1.332 (3)	N1—N2	1.382 (2)
C9—C10	1.500 (3)	N1—H1D	0.8600
C10—C11	1.526 (3)	N2—H2A	0.8600
C10—H10A	0.9700	O1—H1E	0.8200
C10—H10B	0.9700	N3—N3^i^	1.376 (3)
C11—H11A	0.9600	N3—H3B	0.84 (2)
C11—H11B	0.9600	O5—H5B	0.88 (3)
			
C2—C1—H1A	109.5	H11A—C11—H11C	109.5
C2—C1—H1B	109.5	H11B—C11—H11C	109.5
H1A—C1—H1B	109.5	C13—C12—H12A	109.5
C2—C1—H1C	109.5	C13—C12—H12B	109.5
H1A—C1—H1C	109.5	H12A—C12—H12B	109.5
H1B—C1—H1C	109.5	C13—C12—H12C	109.5
C5—C2—C3	118.03 (19)	H12A—C12—H12C	109.5
C5—C2—C1	120.00 (19)	H12B—C12—H12C	109.5
C3—C2—C1	121.96 (19)	C15—C13—C14	118.5 (2)
C4—C3—C2	120.70 (19)	C15—C13—C12	120.2 (2)
C4—C3—H3A	119.6	C14—C13—C12	121.3 (2)
C2—C3—H3A	119.6	C17—C14—C13	120.4 (2)
C3—C4—C7	121.80 (19)	C17—C14—H14A	119.8
C3—C4—H4A	119.1	C13—C14—H14A	119.8
C7—C4—H4A	119.1	C13—C15—C16	121.5 (2)
C2—C5—C6	121.78 (19)	C13—C15—H15A	119.3
C2—C5—H5A	119.1	C16—C15—H15A	119.3
C6—C5—H5A	119.1	O5—C16—C15	120.35 (18)
O1—C6—C5	120.36 (17)	O5—C16—C18	119.40 (18)
O1—C6—C7	119.25 (17)	C15—C16—C18	120.25 (19)
C5—C6—C7	120.39 (18)	C14—C17—C18	121.9 (2)
C4—C7—C6	117.28 (18)	C14—C17—H17A	119.1
C4—C7—C8	117.01 (18)	C18—C17—H17A	119.1
C6—C7—C8	125.70 (18)	C17—C18—C16	117.49 (19)
O2—C8—N1	120.91 (18)	C17—C18—C19	117.19 (18)
O2—C8—C7	122.27 (18)	C16—C18—C19	125.33 (19)
N1—C8—C7	116.81 (17)	O4—C19—N3	120.78 (19)
O3—C9—N2	121.63 (19)	O4—C19—C18	122.20 (19)
O3—C9—C10	122.19 (18)	N3—C19—C18	117.01 (18)
N2—C9—C10	116.18 (18)	C8—N1—N2	120.37 (16)
C9—C10—C11	112.34 (18)	C8—N1—H1D	119.8
C9—C10—H10A	109.1	N2—N1—H1D	119.8
C11—C10—H10A	109.1	C9—N2—N1	119.05 (16)
C9—C10—H10B	109.1	C9—N2—H2A	120.5
C11—C10—H10B	109.1	N1—N2—H2A	120.5
H10A—C10—H10B	107.9	C6—O1—H1E	109.5
C10—C11—H11A	109.5	C19—N3—N3^i^	119.9 (2)
C10—C11—H11B	109.5	C19—N3—H3B	121.4 (16)
H11A—C11—H11B	109.5	N3^i^—N3—H3B	118.6 (16)
C10—C11—H11C	109.5	C16—O5—H5B	110.8 (17)

Symmetry codes: (i) −*x*+2, −*y*+1, −*z*.

### Hydrogen-bond geometry (Å, °)

**Table:**

*D*—H···*A*	*D*—H	H···*A*	*D*···*A*	*D*—H···*A*
N1—H1D···O1	0.86	1.93	2.620 (2)	136
O1—H1E···O4	0.82	1.87	2.682 (2)	169
N2—H2A···O2^ii^	0.86	2.03	2.866 (2)	165
N3—H3B···O5	0.84 (2)	1.94 (3)	2.613 (3)	136 (2)
N3—H3B···O4^i^	0.84 (2)	2.37 (3)	2.655 (3)	101 (2)
O5—H5B···O3^i^	0.89 (3)	1.80 (3)	2.685 (2)	175 (2)

Symmetry codes: (ii) −*x*+1, −*y*+2, −*z*+1; (i) −*x*+2, −*y*+1, −*z*.
